# Direct Peritoneal Resuscitation with Pyruvate Protects the Spinal Cord and Induces Autophagy via Regulating PHD2 in a Rat Model of Spinal Cord Ischemia-Reperfusion Injury

**DOI:** 10.1155/2020/4909103

**Published:** 2020-01-04

**Authors:** Ying Xiong, Yun Xia, Jiangtao Deng, Xuetao Yan, Jianjuan Ke, Jia Zhan, Zongze Zhang, Yanlin Wang

**Affiliations:** ^1^Zhongnan Hospital of Wuhan University, Department of Anesthesiology, Wuhan University, Wuhan 430071, China; ^2^Shenzhen Bao'an Maternity and Child Health Hospital, Department of Anesthesiology, Shenzhen, China

## Abstract

Direct peritoneal resuscitation with pyruvate (Pyr-PDS) has emerged as an interesting candidate to alleviate injury in diverse organs, while the potential mechanism has yet to be fully elucidated. To explore the effect of autophagy in the spinal cord ischemia-reperfusion (SCIR) injury and the underlying mechanism, we established a model of SCIR *in vivo* and *in vitro*. *In vivo*, male SD rats underwent aortic occlusion for 60 min and then followed by intraperitoneally infused with 20 mL of pyruvate or normal saline for 30 min, and the spinal cords were removed for analysis after 48 h of reperfusion. The functional and morphological results showed that Pyr-PDS alleviated SCIR injury; meanwhile, the expression of autophagy-related genes and transmission electron microscopy displayed autophagy was activated by SCIR injury, and Pyr-PDS treatment could further upregulate the degree of autophagy which plays a protective part in the SCIR injury, while there is no significant difference after treatment with saline. In addition, SCIR injury inhibited expression of PHD2, which results to activate its downstream HIF-1*α*/BNIP3 pathway to promote autophagy. In the Pyr-PDS, the results revealed PHD2 was further inhibited compared to the SCIR group, which could further activate the HIF-1*α*/BNIP3 signaling pathway. Additionally, oxygen-glucose deprivation and reoxygenation were applied to SH-SY5Y cells to mimic anoxic conditions *in vitro*, and the expression of autophagy-related genes, PHD2, and its downstream HIF-1*α*/BNIP3 pathway showed the same trend as the results *in vivo*. Besides, IOX2, a specific inhibitor of PHD2 was also treated to SH-SY5Y cells during reoxygenation, in which the result is as same as the pyruvate group. Then, we observed the expression of autophagy-related genes and the HIF-1*α* signal pathway in the process of reoxygenation; the results showed that as the reoxygenation goes, the expression of the HIF-1*α* signal pathway and degree of autophagy came to decrease gradually, while treated with pyruvate could maintain autophagy high and stable through keeping PHD2 at a lower level during reoxygenation, and the latter was observed downregulated during reoxygenation process from 0 to 24 hours in a time-effect way. The above results indicated that direct peritoneal resuscitation with pyruvate showed effective protection to ischemia-reperfusion of the spinal cord through activating autophagy via acting on PHD2 and its downstream HIF-1*α*/BNIP3 pathway.

## 1. Introduction

Thoracoabdominal surgery is accompanied with blockage of blood flow to the aorta, which causes spinal cord ischemia-reperfusion (SCIR) injury, and the latter is a dreaded complication which poses an important challenge to lower the risks of destructive paraplegia and paralysis with high disability rate [1]; the potential mechanism of SCIR injury has lately received great attention [2–4]. Autophagy has attracted extensive interest about the underlying mechanisms in the pathophysiological process of a variety of neurological diseases [5]. Under normal conditions, autophagy maintains at low frequency and conduces to cellular homeostasis, while autophagy can be induced under nutrient starved stress which could degrade cellular component then reuse of recoverable component to reduce cost of survival [6]. It has been proved that autophagy is of great importance in the SCIR injury; nevertheless, it has yet to be identified the effect of autophagy in the development of SCIR injury [7–9]. The intensity and duration of autophagy process are regulated by a variety of autophagy-related proteins such as p62, Beclin-1, and mTOR (mammalian target of rapamycin). Studies have shown that Beclin-1 and mTOR are the downstream of Bcl2/adenovirus EIB19 kDa-interacting protein 3 (BNIP3) which could regulate the extent degree of autophagy in a variety of cells [10, 11].

**Figure 1:**
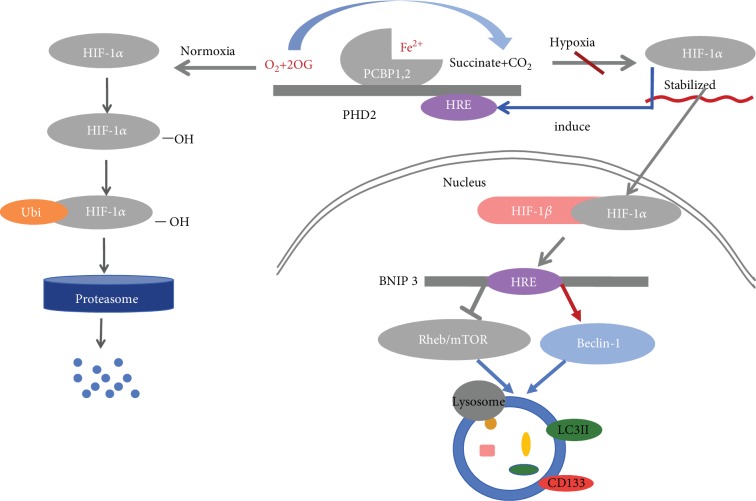
PHDs could hydroxylate HIF-1*α* under normoxia, and the latter is discerned by the von Hippel-Lindau (VHL) tumor suppressor protein and then degraded by the proteasome. Under hypoxic conditions, HIF-1*α* is stabilized due to inactivation of PHDs, and then BNIP3 is upregulated as the direct target of HIF-1*α*. Upregulation of BNIP3 will bind to Rheb which would inhibit mTOR signaling and competing with Beclin-1-Bcl-2 and Beclin-1-Bcl-xl complexes allowing the release of Beclin-1. Autophagy would be enhanced via both sides, eventually, reducing inhibition of autophagy and enhancing activation of autophagy. Meanwhile, under hypoxia, accumulation of HIF-1*α* can induce the transcription of PHD2, in contrast, to ensure swift removal of HIF-1*α* after reoxygenation.

Hypoxia-inducible factor-1 (HIF-1) is the pivotal regulator in hypoxia to get tissues adapted, which could regulate the response to hypoxia and maintain a certain homeostasis of cells and systems by activating transcription of downstream genes [12, 13]. HIF-1 consists of heterodimers including *α*-subunits and *β*-subunits, and the former could be regulated by oxygen levels [14]. Under normal oxygen conditions, HIF-1*α* subunits are tightly regulated by HIF prolyl hydroxylases (PHDs), and then hydroxylated HIF-1*α* could be degraded in a proteasome-dependent way through von Hippel-Lindau (VHL) protein-dependent ubiquitination [15]. According to literature, there are three subtypes of PHD in mammalian, PHD1–3, and PHD2 has a higher affinity to HIF-1*α* under the same conditions. When there are not sufficient quantities of oxygen, the activity of PHD2 is inhibited, and HIF-1*α* would stabilize stem from impeding the degradation [16]. Afterwards, stabilized HIF-1*α* would turn into transcriptional activity via combining with HIF-1*β* and then translocate into the nucleus where the heterodimers would bind to the promoter region, hypoxia reaction element (HRE), of downstream genes, such as BNIP3, in which the promoter region containing HREs is the specific target gene of HIF-1 [17, 18] Figure 1.

Pyruvate plays an important role in the tricarboxylic acid cycle (TCA) and also exerts the effects via anti-inflammatory and antioxidant. Meanwhile, pyruvate is also a key intermediate product in the process of glycolysis, serving as a link hub for the metabolism of carbohydrate, lipids, and amino acids. Studies have shown that pyruvate plays a protective part on hemorrhagic shock and organs with ischemia-reperfusion injury such as the heart, brain, and intestine [19, 20]. Ryou et al. [21] found that pyruvate offered some protection in ischemia-reperfusion injury of cerebral through upregulating the HIF-1 and its downstream genes in both neurons and astrocytes. Whether HIF-1 can do the same in SCIR injury if pyruvate could react on SCIR injury via regulating the expression of HIF-1 has not been reported.

Hence, it is not a surprise that the degree of autophagy has been targeted as a crucial role in studies of SCIR injury. According to the reports, autophagy mediated by HIF-1 is of great importance in the protective effect of pyruvate peritoneal resuscitation on SCIR injury. Up to now, few potential therapeutic target medicines related to autophagy have been applied in the clinic. With further research on the mechanism of SCIR injury, it is hopeful to improve the functional recovery after surgery. In this study, in order to observe the effect of pyruvate and change of autophagy, rats were randomly assigned into 4 groups: the sham group, the SCIR group, the SCIR+saline group, and the Pyr-PDS group. For further illustration, SH-SY5Y cell treatment with oxygen, glucose deprivation, and reperfusion was carried out to investigate the process of reperfusion and underlying mechanisms.

## 2. Method

### 2.1. Chemicals and Reagents

Pyruvate was supplied by Sigma (St. Louis, MO). Reverse transcript and RT-qPCR kits were obtained from ELK Biotechnology (Wuhan, China). Creatinine Assay Kit was obtained from Nanjing Jiancheng Bioengineering Institute (Nanjing, China). TRIpure Reagent was purchased from Aidlab Biotechnologies Co., Ltd. (Beijing, China). Total Protein Extraction Kit was obtained from Aidlab Biotechnologies Co., Ltd. (Beijing, China). BCA Protein Assay Kit was obtained from Aspen Biotechnology (Wuhan, China). Fetal bovine serum (FBS) and MEM/F12 (1 : 1) were supplied by Gibco (St. Louis, MO, USA). Penicillin-streptomycin combination was purchased from Genom (Hangzhou, China). Cell Counting Kit-8 (CCK-8) assay kit was obtained from Abcam (Shanghai, China). Apoptosis Assay Kit Annexin V-FITC-propidium iodide (PI) was purchased from BestBio (Shanghai, China). IOX2 was purchased from MCE (Shanghai, China). All oligonucleotide primers of the rat and human were synthesized by GeneCreate Biological Engineering Co., Ltd. (Wuhan, China). All synthetic concoctions were of analytical grade.

### 2.2. Preparation of Pyr-PDS

In our research, 2.5% Glu-Pyr-PDS (Pyr-PDS; 396 mOsm/L, pH 5.2) was prepared fresh in the laboratory; the concentration of pyruvate is 40 mmol/L, Na^+^ is 132 mmol/L, Ca^2+^ is 1.75 mmol/L, Mg^2+^ is 0.25 mmol/L, Cl^−^ is 96 mmol/L, and glucose is 2.5 g/dL. The pH was changed in accordance with 5.2 with HCl or NaOH. The DPR solutions were stored at 4°C and warmed up to RT before use. The stability of Pyr-PDS was confirmed by the high-performance liquid chromatography [19].

### 2.3. Animals

All the animal procedures were approved by Animal Experiment Committee of Wuhan University (China), and the surgical interventions and postoperative care were conformed to the *Guide for the Care and Use of Laboratory Animals* (National Institutes of Health). A total of 72 male Sprague-Dawley rats weighting 200 to 250 g were purchased from Wuhan University Animal Center in Wuhan, China. Rats were raised in an invariable temperature environment and acclimatized to 12 h light-dark cycles for 1 week. Animals were treated with free access to food and water. Before the experiment, rats were kept with food and water overnight.

### 2.4. The Spinal Cord Ischemia-Reperfusion Injury Model

A total of 72 rats were randomly divided into four groups: sham group, SCIR group, SCIR+saline group, and Pyr-PDS group: 9 rats for Western blot/qPCR, 6 rats for histology, and 3 rats for electron microscope = 18 rats/group × 4 groups = 72 rats. We carried out the SCIR model according to the literature [7, 22]. Briefly, pentobarbital sodium (45 mg/kg, i.p.) was used to get the rats anesthetized. The blood flow of abdominal aorta was blocked above the right renal artery near the heart with a 50 g aneurysm clip for 60 min to establish the SCIR model to ensure Adamkiewicz artery is blocked, which is the main source of blood supply for the spinal cord [23]. In the SCIR+saline and Pyr-PDS groups, 20 mL of saline/Pyr-PDS was intraperitoneally infused with a microinfusion pump over the course of 30 min after the surgery. In the sham group, the abdominal aorta was exposed with no occlusion. The efficiency of the surgery was the evidence if there is neurological deficits in the hind limb. All rats were placed in a box at 28°C to recover then went back to in their cages with free access to food and water.

### 2.5. Neurological Function Assessment

The neurological function deficits after SCIR of rats was evaluated with the Basso, Beattie, and Bresnahan (BBB) open-field locomotor scale [24]. The scores were determined with averaging the values from the three individuals blinded to the groups in 1, 6, 12, 24, and 48 h after reperfusion. A discussion was conducted to reach a consensus when there were disagreements.

### 2.6. Hematoxylin-Eosin Stain of the Spinal Cord

Forty-eight hours after reperfusion, a total of 18 rats (*n* = 6 per group) were anesthetized with excessive pentobarbital sodium and sacrificed by transcardiac perfusion with 100 mL of 0.9% saline solution, and after close therewith is 100 mL of 4% paraformaldehyde in phosphate-buffered saline (PBS), for tissue fixed. Fixed spinal cords were cut into paraffin sections. Sections were dewaxed twice in xylene (5 minutes each). After rehydrating in a series of graded alcohol, sections were rinsed with distilled water for 2 minutes. Afterwards, spinal cord sections were stained with hematoxylin solution for 10 minutes and then 1% acid alcohol for 30 seconds, soaked in running water, and stained with eosin solution for 30–60 seconds again. After rinsing in flowing water for 5 minutes, tissue samples were again dehydrated in a series of graded alcohol (5 minutes each), cleared in xylene (5 minutes each), and mounted with neutral balsam finally. Following HE staining, sections were given a pathological score ranging from 0-6, based on the following: 0 = no damage; 1 = 1‐5 eosinophilic neurons in gray matter; 2 = 6‐10 eosinophilic neurons in gray matter; 3 = more than 10 eosinophilic neurons in gray matter; 4 = less than 1/3 infarction area of gray matter; 5 = 1/3‐1/2 infarction area of gray matter; and 6 = more than 1/2 infarction area of gray matter.

### 2.7. Nissl Staining

Animals were sacrificed as described in the hematoxylin-eosin staining, and then the L4–L6 spinal cord sections (1 mm segments; *n* = 6 per group) were dehydrated with alcohol, and then cleared with xylene, and embedded in paraffin finally. The sections were sliced at 4 *μ*m thickness and were Nissl stained with 1% thionine. After washing, the sections were blocked. The sections were stained at the same time in each experiment. Neurons containing Nissl bodies were counted per section, and to reduce counting bias, neurons counting was performed in every fifth slice by two independent investigators and got the results averaged.

### 2.8. Transmission Electron Microscopy (TEM)

For transmission electron microscopy (TEM) observation, L4–L6 spinal cord samples were harvested as described in the hematoxylin-eosin staining and cut into 1 mm^3^ pieces (*n* = 3 per group). Tissues were fixed in 2.5% glutaraldehyde and 4.0% paraformaldehyde overnight. Fixed tissues were immersed in 1% osmium tetroxide for 2 h, rinsed three times for 5 min. Before staining with 5% uranyl acetate, sections underwent dehydration with gradient acetone and then embedded in Spurr's resin. The images were captured every ten fields of view for every sample to observe autophagosomes and autolysosomes.

### 2.9. Quantitative Real-Time PCR

Forty-eight hours after reperfusion, rats were euthanatized with pentobarbital sodium and then the spinal cord of L4-6 was immediately harvested and stored at −80°C (*n* = 9 per group). Total RNA of the spinal cord tissue and SYSH-5Y cells was isolated using TRIpure Reagent. The total RNA was reversed transcribed using EntiLink™ 1st strand cDNA synthesis kit. Afterwards, cDNA was amplified with EnTurbo™ SYBR Green PCR SuperMix. Primers of rat and human were designed according to the gene sequence showed in Table 1. Data was quantified using 2^−*ΔΔ*Ct^ method with GAPDH for normalization. Reactions were repeated in triplicate.

### 2.10. Western Blot Analysis

Total protein of tissues and SH-SY5Y cell were collected with lysis buffer, and the concentrations of proteins were measured. Aliquot proteins were separated on 10% SDS-PAGE and electroblotted onto polyvinylidene difluoride membranes. The membrane was blocked with 5% nonfat dried milk for 2 h, subsequently, incubated with primary antibody overnight at 4°C. Related antibodies were as follows: rabbit anti-GAPDH (1 : 10000, Abcam), rabbit anti-PHD2 (1 : 500, Novusbio), rabbit anti-p62 (1 : 2000, Abcam), rabbit anti-Beclin-1 (1 : 1000, CST), rabbit anti-LC3 (1 : 1000, CST), and horseradish peroxidase (HRP) (1 : 10000, Aspen).

### 2.11. Cell Culture and Oxygen-Glucose Deprivation

Human SH-SY5Y cells, derived from human hippocampal neurons, were obtained from iCell Bioscience (Shanghai, China). Cells were cultured at 37°C in 5% CO_2_ in MEM/f12 medium supplemented with 10% FBS and 1% penicillin-streptomycin. To mimic spinal cord ischemia injury in vitro, we established the model of oxygen-glucose deprivation and reperfusion (OGD/R) as literature described [25, 26]. Briefly, cells were cultured in glucose-free DMEM and incubated at 37°C in hypoxia for 2 h, which was 1% O_2_, 94% N_2_, and 5% CO_2_. For reoxygenation, the culture medium was replaced by DMEM medium with pyruvate (100 *μ*M) and cells were put into the incubator at 37°C and in normoxia for 0, 6, 12, and 24 h. Cells were treated in 5 ways: (i): control group; (ii): OGD group (SH-SY5Y cells were treated with deprivation of oxygen-glucose without reoxygenation); (iii): OGD/R group (SH-SY5Y cells were treated with reoxygenation following oxygen-glucose deprivation); (iv): the pyruvate group; and (v): PHD2 inhibitor group: IOX2 group.

### 2.12. Cell Viability Assay

We used Cell Counting Kit-8 (CCK-8) assay to do cell viability analysis according to the provider's instructions. In brief, cells were seeded into a 96-well plate at 5 × 10^3^ cells/well in 100 *μ*L culture medium overnight (37°C, 5% CO_2_). Afterwards, cells were treated with OGD for 2 h; for reoxygenation, different concentrations of pyruvate (0, 10, 100, 1000 *μ*M) and IOX2 (0, 10, 100, 500 *μ*M) were added to culture medium. Subsequently, 10 *μ*L of the CCK-8 solution was carefully added to 96-well plate and then incubated for 2 h at 37°C. The absorbance was detected at 570 nm with a microplate reader.

### 2.13. Annexin V Staining and Flow Cytometry

We detected the percentage of apoptotic cells insulted by OGD with Annexin V-FITC/PI Detection Kit according to the manufacturer's instructions. In short, cells were digested with EDTA-free trypsinization and then rinsed twice with cold PBS. After resuspended in 400 *μ*L binding buffer, Annexin V (5 *μ*L) and PI (10 *μ*L) were added and then kept on the ice for 15 min in darkness, and then apoptosis rate of SH-SY5Y cells was detected by flow cytometry.

### 2.14. Statistical Analysis

We performed the calculations by Prism (GraphPad Software, La Jolla, CA, USA). Quantitative data were expressed as the mean ± S.E.M. of three independent experiments, normalized to controls. Bartlett's test was used to evaluate equal variances, and one-way analysis of variance (ANOVA) followed by the Student-Newman-Keuls test was used for statistical validations (^∗^*p* < 0.05, ^∗∗^*p* < 0.01, and ^∗∗∗^*p* < 0.001).

## 3. Result

### 3.1. Direct Peritoneal Resuscitation with Pyr-PDS Attenuated Ischemia-Reperfusion-Induced Spinal Cord Injury

**Figure 2:**
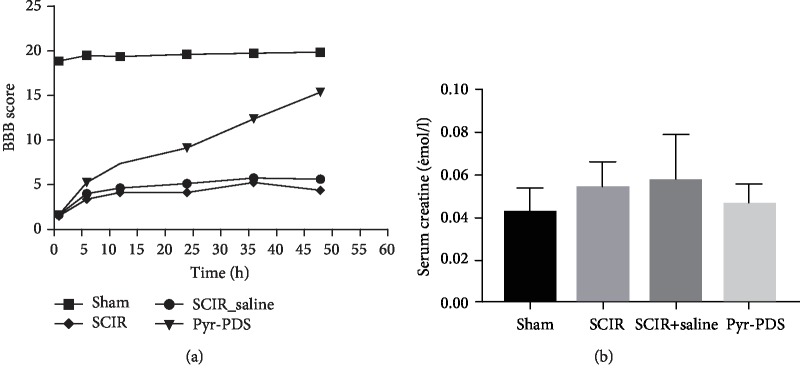
Pyr-PDS showed protective effect after SCIR. (a) Pyr-PDS improved BBB score after SCIR injury at 1 h, 6 h, 12 h, 24 h, 36 h, and 48 h post injury. (b) Serum creatinine showed SCIR has no influence to kidney function. Data are shown as the mean ± S.E.M., *n* = 18.

**Figure 3:**
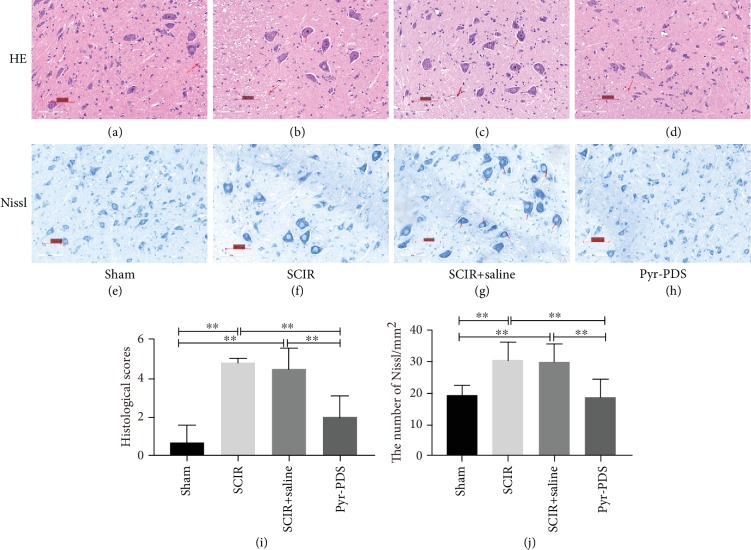
Direct peritoneal resuscitation with pyruvate alleviates SCIR injury and Nissl staining. (a–d) Representative section for hematoxylin-eosin staining (HE) staining at 48 hours after injury. Scale bar 200 *μ*m for magnification 20x. (e–h) Nissl staining to display the survival neurons. Scale bar 200 *μ*m for magnification 20x. (i, j) Spinal cord injury histological scores and statistics of the Nissl staining results. Data are shown as mean ± S.E.M.; ^∗^*p* < 0.05, ^∗∗^*p* < 0.01 vs. control, *n* = 6.

According to the research related to the injury of the spinal cord, paralysis may ensue in both hind limbs after SCIR injury. To assess the function of pyruvate-based peritoneal dialysis with pyruvate solution (Pyr-PDS) on ischemia-reperfusion-induced spinal cord injury, we assessed locomotor function with the BBB locomotor test, a standard method to evaluate for function recovery after SCIR in rats. As shown in Figure 2, rats treated with or without SCIR showed significantly lower BBB score compared to the sham group, which is considered that the SCIR model was successfully established in rats. Also, function of hind limb locomotor recovered gradually over time throughout the evaluation period in the Pyr-PDS group than the SCIR group or the SCIR+saline group at six point-in-time from 1 to 48 hours post injury, which indicates protective effect of Pyr-PDS. Additionally, as shown in Figure 2(b), there is no statistic difference of serum creatinine among the four groups which means surgery has no influence to renal function. Besides, as a result in Figure 3, HE staining (a–d) and Nissl staining (e–h) (×20) were performed to study the morphological changes of the spinal cord at 48 h after reperfusion. HE staining revealed that neurons in the group of SCIR with or without saline were both swelling; there were no clear borderline and vacuolation that were also between white matter and gray matter, while the injury has been improved a lot in the Pyr-PDS group. Likewise, Nissl staining showed that in the SCIR group and SCIR+saline group, Nissl bodies accumulated in the cytoplasm and were darkly stained compared to the sham group, which is not apparent in the Pyr-PDS group. In general, it is obvious that spinal cord injury induced by I/R was prominently attenuated by Pyr-PDS other than saline in both functional and morphological results.

### 3.2. Pyr-PDS Increased Autophagy after SCIR Injury

**Figure 4:**
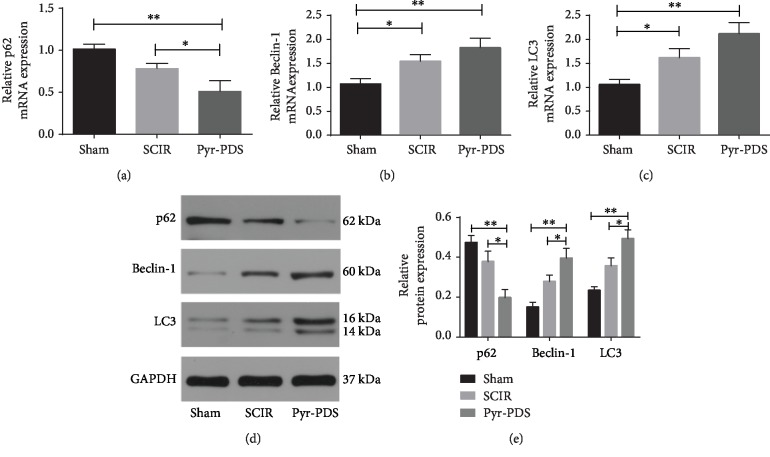
Pyr-PDS increased expression of mRNA and protein related to autophagy in the spinal cord. The mRNA expression levels of p62 (a), Beclin-1 (b), and LC3 (c) were determined by qPCR. The expression levels of autophagy-related protein were detected by Western blot (d), and summarized data of protein expression was exhibited (e). Data are shown as the mean ± S.E.M.; ^∗^*p* < 0.05, ^∗∗^*p* < 0.01*vs*. control.

**Figure 5:**
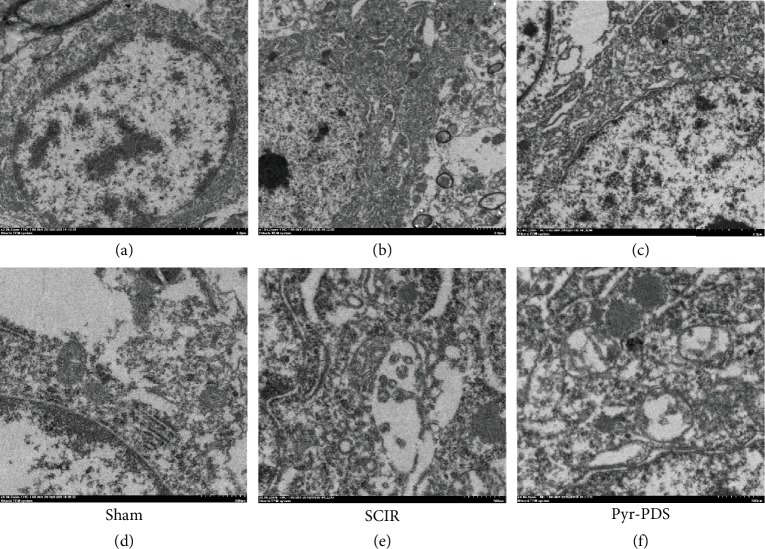
SCIR could activate autophagy compared to the sham group, and Pyr-PDS could further increase degree of autophagy. Transmission electron microscopy (TEM) exhibited fragmented mitochondria and autophagosomes with double membranes which were marked with black arrowheads. (a–c) Scale bar = 2 *μ*m; (d–f) Scale bar = 500 nm.

To investigate whether Pyr-PDS exerted protective effect through regulating autophagy in SCIR injury, we observed autophagosome and the expression of autophagy-related genes and protein (as there is no significant difference in the SCIR+saline group according to both functional and morphological results, we only compared the difference of autophagy among the sham group, SCIR group, and Pyr-PDS group.). As data showed in Figure 4, the results exhibited that at 48 h after reperfusion, expression in levels of both mRNA and protein of LC3 and Beclin-1 in the spinal cords of the SCIR group was increased than the sham group, while treatment with Pyr-PDS could further increase the expression in levels of mRNA and protein of autophagy. The expression of p62 is opposite, though. Besides, the autophagosomes were evaluated with TEM in Figure 5, which was utilized to identify the morphological autophagosomes with double membranes. We can observe the regular mitochondria, endoplasmic reticulum, and regular myelinated axons in the spinal cord cells and surrounding myelin sheaths in the sham group. In contrast, disordered structure of mitochondria, myelinated axons, numerous lysosomes with a double-membrane structure, and parts of cytoplasmic organelles and nuclear pyknosis had occurred in the SCIR group, while after treated with Pyr-PDS, the injury was alleviated and there were more autophagosomes and autolysosomes compared with the SCIR group.

### 3.3. Direct Peritoneal Resuscitation with Pyr-PDS Increased Autophagy in SCIR by Upregulating the HIF-1*α*/Bnip3 Pathway

**Figure 6:**
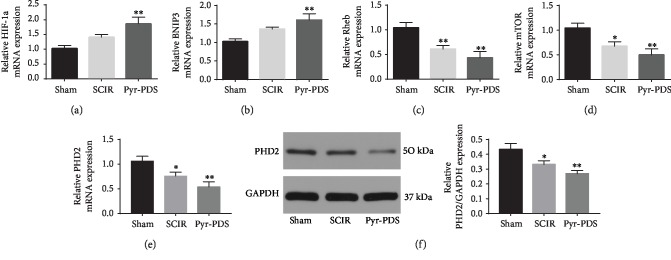
SCIR increased mRNA expression of the HIF-1*α* signal pathway, which could be further upregulated after Pyr-PDS treatment. (a–d) mRNA expression of HIF-1*α*, BNIP3, Rheb, and mTOR. (e) mRNA expression of PHD. (f) Protein expression of PHD2 and summarized data of protein expression. Data are shown as the mean ± S.E.M.; ^∗^*p* < 0.05, ^∗∗^*p* < 0.01 vs. control.

To investigate the underlying mechanism of autophagy on the effect of pyruvate in SCIR, we performed analysis of mRNA expression of HIF-1*α* which is of great importance to induce autophagy in the ischemia and reperfusion, which was upregulated in the SCIR group compared with the sham group. Ulteriorly, the mRNA expression of HIF-1*α* was further increased treated with Pyr-PDS compared to the SCIR group. In addition, as an identified downstream gene regulated by HIF-1*α*, Bnip3 exerted essential effect in the HIF-1 signaling pathway in numerous studies. Our research explored the effect of the HIF-1*α*/Bnip3 signaling pathway in regulating autophagy induced by SCIR. As shown in Figure 6, the upregulation of the mRNA expression of Bnip3 was observed in the SCIR group; the mRNA expression of Bnip3 was upregulated to a greater extent in the Pyr-PDS group which is as the same varying trend as HIF-1*α*. To better know the mechanism of the HIF-1*α*/Bnip3 pathway alteration in the induction of autophagy, we analyzed the expression of Rheb and its downstream gene mTOR. The data shows decrease in both Rheb and mTOR mRNA expressions in the SCIR group and further reduced in the Pyr-PDS group, suggesting that activation of autophagy induced by the HIF-1*α*/Bnip3/Rheb/mTOR pathway in the SCIR group gets involved in the injury induced by ischemia and reperfusion. In the meantime, as shown in Figure 4(b), beclin-1, a pivotal regulator in the process of autophagy which could be regulated by Bnip3, is increased in the SCIR group and further upregulated in the Pyr-PDS group, implying that increase of autophagy induced by the HIF-1*α*/Bnip3/beclin-1 pathway in SCIR injury is also of great significance. In addition, expression of PHD2 at mRNA and protein level was also detected, and the result shows that expression of PHD2 was inhibited by SCIR, and it could be further decreased by Pyr-PDS.

### 3.4. Pyruvate Improved Injury of SH-SY5Y Cells Induced by OGD Treatment via Activating Autophagy

**Figure 7:**
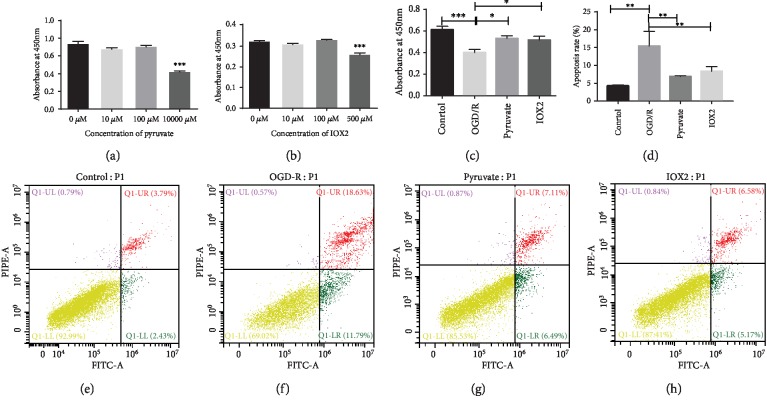
Pyruvate/IOX2 could attenuate injury induced by OGD/R in SH-SY5Y cells. (a) The result of CCK-8 showed 1000 *μ*M of pyruvate was cytotoxic in comparison to others. (b) The result of CCK-8 showed 500 *μ*M of IOX2 was cytotoxic in comparison to others. (c) Both pyruvate and IOX2 play a protective role of SH-SY5Y treated by OGD/R according to the result of CCK-8. (e–h) Flow cytometry displays the apoptosis rate is increased in the OGD/R group, while downregulated by pyruvate/IOX2, and summarized data of flow cytometry was exhibited (d). Data are shown as the mean ± S.E.M.; ^∗^*p* < 0.05, ^∗∗^*p* < 0.01, ^∗∗∗^*p* < 0.001*vs*. control.

To further research the potential mechanisms of pyruvate alleviating spinal cord injury induced by I/R, OGD/R treatment was carried out to mimic ischemia-reperfusion injury in vitro in hippocampal neuron cell line, SH-SY5Y cells. As shown in Figures 7(a) and 7(b), CCK-8 was performed to explore the appropriate concentration of pyruvate (100 *μ*M) and IOX2 (100 *μ*M) which showed no toxic effect to SH-SY5Y cells. Afterwards, pyruvate (100 *μ*M) and IOX2 (100 *μ*M) were given to SH-SY5Y cells after treated with OGD/R; the result in Figure 7(c) showed a significant increase in the group treated with pyruvate and IOX2 compared to the OGD/R group. Additionally, the apoptotic rate of SH-SY5Y cells was detected by flow cytometry, which was markedly increased after treatment with OGD/R compared to the control group (*p* < 0.01), whereas treatment with pyruvate and IOX2 significantly downregulates the apoptotic rate compared with OGD/R treatment. The results of flow cytometry indicated treatment with pyruvate and IOX2 exerted same protective role in the OGD/R injury in vitro.

### 3.5. Pyruvate Activated Autophagy in OGD/R-Induced Injury in SH-SY5Y Cells

**Figure 8:**
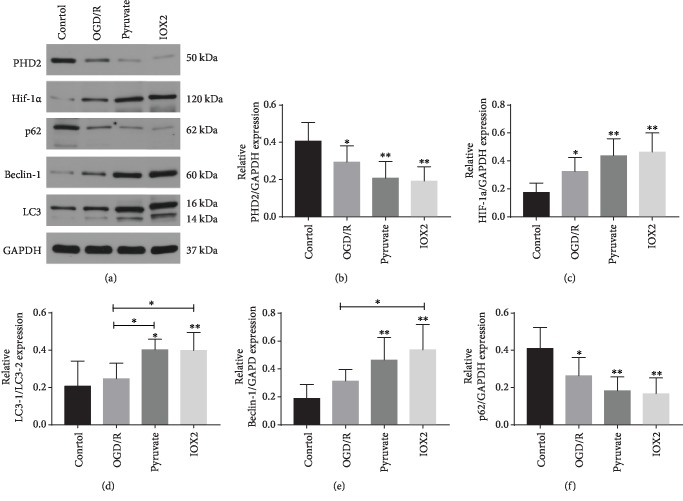
SH-SY5Y cells treated with OGD/R higher expressed autophagy-related proteins, HIF-1*α* and PHD2 (a), which could be further increased by pyruvate/IOX2 treatment. Summarized data of protein expression was also exhibited (b–f). Data are shown as the mean ± S.E.M.; ^∗^*p* < 0.05, ^∗∗^*p* < 0.01 vs. control.

To explore whether autophagy gets involved in the protective effect of pyruvate against hypoxia-reperfusion *in vitro*, we observed autophagy-related protein expression and its upstream PHD2 and HIF-1*α*. As we can see in Figure 8, autophagy is activated in the OGD/R group, which was further upregulated after treated with pyruvate. Also, expression of PHD2 was inhibited in the OGD/R group, and pyruvate could further decrease protein level of PHD2. HIF-1*α* shows increasing expression in OGD/R, and higher expression of HIF-1*α* was observed in pyruvate. Furthermore, cells treated with IOX2 which is a specific inhibitor of PHD2 show the same result as the pyruvate group; the result confirmed that pyruvate exerts protective effect through inhibiting PHD2.

### 3.6. Reoxygenation Could Upregulate Expression of PHD2 and the HIF-1*α* Signal Pathway as the Oxygen Concentration Goes Up Which Could Be Reversed by Pyruvate

**Figure 9:**
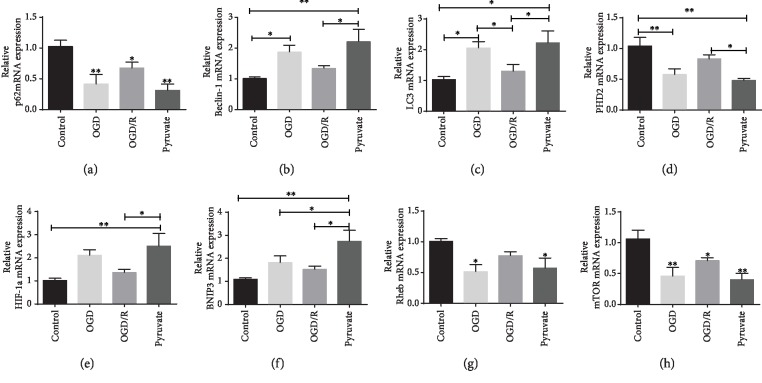
Reperfusion could reverse the increase expression of autophagy-related genes (a–d) and the HIF-1*α* signal pathway (e–h) in mRNA level in SH-SY5Y cells compared with the OGD group, while pyruvate could reverse the decline of the HIF-1*α* pathway and autophagy-related mRNA expression during reperfusion. Data are shown as the mean ± S.E.M.; ^∗^*p* < 0.05, ^∗∗^*p* < 0.01 vs. control.

**Figure 10:**
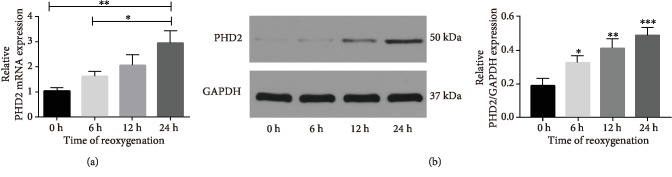
We detected the expression of PHD2 in both mRNA and protein levels of SH-SY5Y cells, as displayed in (a) the expression of PHD2 is decreased in SH-SY5Y cells treated with OGD; the expression of PHD2 was further downregulated in the SH-SY5Y cells in the group of pyruvate, while as shown in (b), the results exhibited the expression of PHD2 of SH-SY5Y cells treated with OGD is increased with the time of reoxygenation. Statistical significance was considered difference with ^∗^*p* < 0.05, ^∗∗^*p* < 0.01 vs. control.

As displayed in Figure 9, the mRNA level of HIF-1*α* and Bnip3 was increased in the OGD group, which was decreased in the OGD/R group, though. While treatment with pyruvate after OGD/R, the mRNA level of HIF-1*α* and Bnip3 was upregulated again. Additionally, as downstream genes of Bnip3, the expression of Rheb and its downstream gene mTOR was analyzed; both Rheb and mTOR expressions were upregulated treated with OGD and decreased with reoxygenation going; while in the pyruvate group, there is a fewer level of Rheb and mTOR mRNA expression. Results mentioned above indicated that increase of autophagy induced by the HIF-1*α*/Bnip3 pathway was of great significance in the OGD group, which worked through regulating Rheb, mTOR, and Beclin-1. Moreover, we observed pathway changed over the process of reoxygenation and reversed by pyruvate. The results indicated that the HIF-1*α*/Bnip3 pathway was activated to upregulate the degree of autophagy after OGD *in vitro*, while the process of reperfusion reversed the activation of the HIF-1*α*/Bnip3 signal pathway, which could be increased again by pyruvate treatment. Additionally, as shown in Figure 9, expression of PHD2 decreased in the OGD group compared to the control, and it increased after reperfusion in the OGD/R group, while pyruvate treatment could reverse the increase of PHD2 expression over reperfusion process. Then, we observed the expression over the reperfusion from 0 to 24 hours, as shown in Figure 10; both mRNA and protein expressions of PHD2 are related to the time of reoxygenation, which means the activity of PHD2 is inhibited under hypoxia; as the oxygen concentration increases, the activity of PHD2 improves gradually, while pyruvate could inhibit the increase of PHD2 on the basis of the reperfusion.

## 4. Discussion

With high morbidity and disability rate, SCIR is not only a threat to patients' life but also a great burden to families and society. According to the pathophysiological characteristics, SCIR injury is divided into two stages, the early stage of acute ischemic injury and the late stage of reperfusion injury. The ischemic stage followed by decreased of blood flow leads to hypoxia of the spinal cord with accumulation of metabolic product, including lactic acid, hypoxanthine, and lipid peroxides. At the reperfusion stage, restored blood flow brought a large amount of oxygen which consumed to produce uric acid and a large number of free radicals which could generate lipid peroxides via interacting with lipids and mitochondrial membranes; as a result, excess lipid peroxides would further lead to cell death. This process is defined as “reperfusion injury” [27]. There are lots of pathologic events participating in the SCIR injury, including the lack of ATP, reduction of pH intracellular, and mitochondrial calcium overload caused by the ischemia. During reperfusion, oxygen paradox and endoplasmic reticulum stress could aggravate the injury [28]. Nevertheless, few progress has been made to explore the therapeutic strategies of ischemia-reperfusion of spinal cord injury. Previous research has demonstrated pyruvate protected splanchnic organ from ischemic injury through effective antioxidant and free radical scavenger [19]. Also, recent studies [19, 29]showed that direct peritoneal resuscitation could alleviate the ischemia injury through improving the organ blood flow and reducing organ edema and tissue necrosis. With this clinical requirement in mind, we observed whether there is protective effect of SCIR injury treated with direct peritoneal resuscitation with Pyr-PDS. Here, results of function and morphological observations revealed that neurons in the SCIR group were damaged, while BBB score was increased gradually in the Pyr-PDS group and same as morphological results. It was indicated that direct peritoneal resuscitation with Pyr-PDS exerts neuroprotective effects in rats with SCIR injury. While there is no difference between the SCIR group and SCIR+saline group, the interference of intraperitoneal injection was excluded. Considering complication to surrounding organs, renal function was evaluated. Though there is no statistic difference of serum creatinine among the four groups, trend that serum creatinine is higher in the SCIR/SCIR+saline group, and Pyr-PDS decreases the level of serum creatinine existed, which might indicate that although the right renal artery was blocked, functional compensation of left renal might be the reason that there is no significant change of renal function; also, treatment with Pyr-PDS may also play a protective part in the renal injury, which could be further investigated. Besides, *in vitro*, both results of flow cytometry and CCK-8 indicated that pyruvate and IOX2 played a protective role in the cells treated with OGD/R. Above all, our results demonstrated pyruvate exerted protection against ischemia-reperfusion injury of the spinal cord both *in vivo and vitro*; in the meantime, it is an effective method to improve SCIR injury by direct peritoneal resuscitation with Pyr-PDS. Furthermore, as for direct peritoneal resuscitation, direct peritoneal resuscitation increases the perfusion of the spinal cord by a related mechanism based on the local vascular endothelium compared to the intravenous administration. Besides, based on the previous study [19], there are changes in peritoneal solute transport resulting from pyruvate treatment, accompanied by a reduction in both peritoneal membrane angiogenesis and fibrosis, indicating novel mechanisms that could potentially reduce glucose-driven alterations to the peritoneal membrane *in vivo*. Moreover, compared to direct peritoneal resuscitation, intraperitoneal resuscitation is also reported to be a good way to regulate microcirculation, protect endothelial cells, and reduce third spacing of fluid, which was reported that it can ameliorate visceral vasoconstriction following intravenous resuscitation (VR). Based on this, different effects of intraperitoneal resuscitation and direct peritoneal resuscitation could be further explored in the future.

The degree of the cell injury depends on whether cells could adapt the redox environment and oxidative stress during ischemia and reperfusion. To get better adapted to change of milieu interne, autophagy would be activated to degrade damaged organelles, toxic agents, and unwanted proteins which could regulate intracellular catabolism. While the degree of autophagy activation is crucial for its role in the development of diseases [19], which seems like a sword with double blades, whether it exerts protection or damage in the pathological process of SCIR injury remains controversial. Due to high energy demand and distinctive morphology, strict autophagy efficiency is very necessary to neurons [30], whether autophagy could protect the spinal cord from injury following I/R remains to be elucidated. Autophagy could be regulated throughout the whole process from formation to degradation of autophagosome. For example, it has been widely confirmed mTOR could inhibit autophagy by regulating the initiation and formation of autophagosomes [31]. On the contrary, Beclin-1 is homologous genes of Atg6/vacuole in yeast protein separation (Vps-30) protein, which can promote the extension of lipid membrane, recruiting superfluous proteins and organelles in cells, and promote autophagosome maturity [32]. As shown in the result, both mRNA and protein expressions related to autophagy and TEM exhibited the number of autophagosomes showed autophagy in SCIR is upregulated and further increased in the Pyr-PDS group. So, we suppose that the underlying mechanism of protective Pyr-PDS may be connected with the activation of autophagy. Meanwhile, according to a systematic review and meta-analysis, SH-SY5Y cell line was the most commonly used tool in human in vitro ischemic research [33] which was also reported to be used to mimic spinal cord injury in vitro [34]. Therefore, SH-SY5Y cells were chosen to be treated with OGD to mimic ischemia and OGD/R to mimic ischemia-reperfusion process in this study. Results showed that autophagy was activated in cells treated with OGD, while autophagy came to decrease during reoxygenation. Of interest, cells treated with pyruvate and IOX2 reversed decline of autophagy during reperfusion, which exhibited higher degree of autophagy compared to the OGD/R group. Videlicet, autophagy is activated at the beginning of hypoxia, and it comes to reduce with time of reperfusion, whereas treatment with pyruvate/IOX2 protects cells from OGD/R injury through reserving the decrease of autophagy during the process of reperfusion. Of interest, there is no difference between the results of IOX2 and pyruvate, which means pyruvate could play a protective role via inhibiting PHD2. In general, we conclude that activation of autophagy exerts protective effect during SCIR injury both *in vivo* and *in vitro*. Additionally, several studies concluded that inhibition of autophagy could relieve the injury of neurological function, which was opposite with our conclusion. As mentioned above, autophagy acts like a double sword; under stress condition, it can be induced which could degrade cellular component then reuse recoverable component to reduce cost of survival then exerts a protective effect. Once autophagy is overactivated, autophagic cell death would lead to tissue damaged. The final outcome depends on the intensity and duration of autophagy process, while the degree of autophagy activation is varied with the process of spinal cord ischemia-reperfusion. As reported [35], the early activated autophagy alleviates spinal cord I/R injury via inhibiting apoptosis and inflammation; however, the later excessive elevated autophagy aggravates I/R injury through inducing autophagic cell death. Furthermore, according to Fan et al. [36], autophagy plays a protective role in that ischemia and ischemic preconditioning protects spinal neurons by sustaining autophagy. In this research, we concluded that autophagy was activated in the process of ischemia, while it came to decrease as the reperfusion went. Decreased autophagy in the early stage of ischemia-reperfusion injury is not enough to reduce cost of cell survival. So, it is of great importance to keep autophagy at a certain intensity; that is the reason pyruvate could be a protector through increasing autophagy. Actually, the role of autophagy is also affected by extent of spinal cord injury, and the latter depends on whether blood flow of the spinal cord was fully blocked, duration of blocking blood flow, and so on. In our study, to stop the blood flow of the spinal cord, we blocked above the right renal artery near the heart to ensure Adamkiewicz artery is blocked, which was reported to be much larger than any other principal artery, supplies a considerable part of the spinal cord, and is therefore of great functional importance which is mostly invariably present between T10 and L2 [23]. However, there is some collateral circulation originated from the subclavian artery or the anastomotic branches of the thoracic and abdominal arteries and the iliac arteries which could supply blood to the spinal cord. Due to the blood supply of the spinal cord was not completely blocked, the injury is not so severe that autophagy was not excessively activated, which is consistent with research that early elevated autophagy protects the spinal cord; however, the later excessive autophagy exerts destructive effects [35]. That is the reason we conclude promoting autophagy has a neuroprotective effect. In brief, the extent and persistence of autophagy activation may be the critical factor to explain the opposing effects to SCIR injury.

Several studies show that iron, 2-oxoglutarate (2-OG), and oxygen are necessary for efficient function of PHDs [37]. Under hypoxia, the activity of PHDs is inhibited, which leads to HIF-1*α* proteins stabilized and further translocates into nuclear and promotes the expression of downstream genes, such as BNIP3 [11]. In the studies of myocardial ischemia-reperfusion, HIF-1 has been shown to alleviate ischemia-reperfusion injury by inducing protective metabolism in myocardial cells [38]. According to the literature, upregulation of BNIP3 can induce autophagy in two different ways: one is competing with Beclin-1/Bcl-2 and Beclin-1/bcl-xl complexes which leads to Beclin-1 release and then enhances autophagy [19, 39] and another is it directly binds to Rheb and decreases GTP-bound Rheb which is active and then reduces the expression of mTOR to alleviate the inhibition of autophagy. Rheb is one of the small Ras GTPase superfamilies, and BNIP3 downregulates GTP-bound Rheb via binding to Rheb [40, 41]. Furthermore, diverse studies have demonstrated Rheb is a key activator of mTOR, which blocks autophagy at the posttranslational and transcriptional levels [42]. Due to the functional result, morphological result, and expression of autophagy-related mRNA and protein, the SCIR+saline group showed no significant difference compared to the SCIR group; we concluded that direct peritoneal resuscitation with saline could not alleviate SCIR injury. Then, the expression of the HIF-1*α*/BNIP3 signal pathway was compared among the sham group, SCIR group, and Pyr-PDS group. In the SCIR group, the data shows the HIF-1*α*/BNIP3 pathway and the downstream are increased and the genes that inhibit the autophagy are lower. While in the Pyr-PDS group, the HIF-1*α*/BNIP3 pathway and its downstream that promotes autophagy are further upregulation and genes that inhibit the autophagy are further reduced. For further research variation of the autophagy in the SCIR injury, we observed SH-SY5Y cells treated by OGD and OGD/R. *In vitro*, cells treated with OGD showed the HIF-1*α*/Bnip3 pathway and the downstream genes were increased and the genes that inhibit the autophagy are decreased which is as same as the trend *in vivo.* Differently, we observed the process of reoxygenation; the results showed that the HIF-1*α*/Bnip3 pathway and its downstream which promote autophagy came to reduce gradually; the genes that inhibit autophagy are upregulation as the cell continued reoxygenation. Interestingly, cells treated with pyruvate in the progress of reoxygenation can reverse the expression reduction of the HIF-1*α*/Bnip3 signaling pathway and its downstream thus to activate autophagy and keep autophagy at a certain intensity. Moreover, according to the hypothesis of this research, only the protein expression HIF-1*α* should be upregulated due to the blocking of degradation. While we also observed the increase expression of HIF-1*α* in the level of mRNA, which may be related to the influence of pyruvate to inflammation/ROS in the process of ischemia-reperfusion, the latter were reported to associate with the transcription of HIF-1*α* [43]. All in all, it should be further researched to figure out the underlying mechanism of the different expressions of HIF-1*α* in transcriptional level.

As mentioned above, iron, 2-oxoglutarate (2-OG), and oxygen are of great necessary to efficient function of PHDs; pyruvate could bind to 2-OG site of PHDs and inhibit it thus stabilized HIF-1*α* and its downstream genes; the key point that pyruvate regulates the HIF-1*α*/Bnip3 pathway and its downstream is interaction with PHD2 [44, 45]. As data shows, the protein expression of PHD2 is decreased in hypoxia, during reoxygenation; the expression of PHD2 is kept rising due to elevated concentration of oxygen and then leads to inhibit the activation of HIF-1*α* and its downstream signal pathway which regulates the degree of autophagy. While treatment with pyruvate when reoxygenation resulted in sustaining reduction of PHD2 expression and thus maintained the expression of HIF-1*α* at a higher and more steady level rather than increased PHD2 expression with concentration oxygen went up which was induced by the negative feedback between HIFs and PHDs: under hypoxia, HIF-1*α* accumulated can induce the transcription of PHD2, in contrast, to ensure swift removal of HIF-1*α* after reoxygenation [46, 47]. That is to say, the upregulation of PHD2 during reperfusion is to avoid excessive stress of tissue cells on hypoxia; nevertheless, the negative feedback mechanism is somewhat so protective that tissues suffered I/R do not maximize the potential to adapt the injury; pyruvate acts as a pusher through maintaining a flat line of the activity PHD2 in the process of reoxygenation. Past studies have not focused on the change of PHD2 in the process of reperfusion during ischemia-reperfusion injury, which may be a new therapeutic target of SCIR injury and a new aspect of Pyr-PDS to protect tissues from I/R injury.

## 5. Conclusions

This study supported the evidence that DPR with Pyr-PDS alleviates SCIR injury significantly via activating autophagy both in *vivo* and *vitro*, and the mechanism may be related to interaction between pyruvate and PHD2. Pyruvate could reverse the decline of PHD2's activity during reoxygenation and maintain the activity PHD2 a flat line. The latter regulates the level of the HIF-1*α*/Bnip3 signal pathway which could affect the degree of autophagy by promoting Beclin-1 released through competing with Beclin-1/Bcl-2 and Beclin-1/bcl-xl complexes or directly binding to Rheb to reduce the expression of mTOR. Our research indicated that DPR with Pyr-PDS exerted protection against SCIR injury and pyruvate exerted protection through inhibiting PHD2 and promoting the HIF-1*α*/BNIP3 pathway and autophagy. The study on SCIR injury can provide new drug target for clinical prevention and treatment.

### 5.1. Limitation

In this study, SH-SY5Y cells were chosen to mimic the SCIR model in vitro due to it was reported to be the most commonly used tool in human in vitro ischemic research, while that was the truth that there exists difference between SH-SY5Y cell lines and the spinal cord, and it will be much more convictive if primary spinal cord neuron cells were chosen to confirm our conclusion furtherly. Besides, as mentioned in discussion, we have already proved the protective effect of Pyr-PDS, while there are two points that need further research: (1) we observed that the influence of pyruvate may be related to inflammation/ROS in the process of ischemia-reperfusion due to not only protein level but also mRNA level of PHD2 level was upregulated, to obtain more potent evidences of Pyr-PDS's effects; the influence of inflammation/ROS should also be explored. (2) There is trend that renal function is improved by Pyr-PDS, which might indicate that treatment with Pyr-PDS may also play a protective part in the injury of abdominal organs, which could be further investigated.

## Figures and Tables

**Table 1:** Primers used in this study.

Name		Nucleotide sequence of primers (5′-3′)	Size (bp)
H-HIF-1a	Sense	CCGATGGAAGCACTAGACAAAGT	285
	Antisense	TTTGAGGACTTGCGCTTTCAG	
H-mTOR	Sense	TCAGCCTGTCAGAATCCAAGTC	196
	Antisense	TTGAAGATGAAGGTGATGGCC	
H-BECN1	Sense	GGCACAATCAATAACTTCAGGC	203
	Antisense	GGCAGCTCCTTAGATTTGTCTG	
H-LC3II	Sense	TCCGACTTATTCGAGAGCAGC	167
	Antisense	AGCATTGAGCTGTAAGCGCC	
H-BNIP3	Sense	CGTTCCAGCCTCGGTTTCTA	287
	Antisense	TATTTTCCGGCCGACTTGAC	
H-RHEB	Sense	GACCTGCATATGGAAAGGGTG	199
	Antisense	AGCAGAATCACATCACCGAGC	
H-LC3I	Sense	ACCGCTGTAAGGAGGTACAGC	281
	Antisense	GAAGCCGTCCTCGTCTTTCT	
H-p62	Sense	GAACAGATGGAGTCGGATAACTG	232
	Antisense	CTGGGAGAGGGACTCAATCAG	
H-phd2	Sense	TACGCCACTGTAACGGGAAG	235
	Antisense	GGTTCAATGTCAGCAAACTGG	
H-GAPDH	Sense	CATCATCCCTGCCTCTACTGG	259
	Antisense	GTGGGTGTCGCTGTTGAAGTC	
R-Rheb	Sense	AGCAGGGCAGGATGAATATTC	161
	Antisense	TAATCGGTATCTGCACTTTCCC	
R-MTOR	Sense	TAATGACGAGACCCAGGCTAAG	122
	Antisense	ATGCTCCATCGTGAGTATCCC	
R-HIF-1*α*	Sense	AAGCCCAGAGTCACTGGGACT	118
	Antisense	GTACTCACTGGGACTGTTAGGCTC	
R-beclin-1	Sense	TCAATGCGACCTTCCATATCTG	222
	Antisense	CTGTCAGGGACTCCAGATACGAG	
R-GAPDH	Sense	CGCTAACATCAAATGGGGTG	201
	Antisense	TTGCTGACAATCTTGAGGGAG	
R-LC3-I	Sense	CGAGTTGGTCAAGATCATCCG	138
	Antisense	CCGTCTTCATCCTTCTCCTGTT	
R-LC3-II	Sense	GAGTGGAAGATGTCCGGCTC	229
	Antisense	GACACACTCACCATGCTGTGC	
R-BNIP3	Sense	ATGGGATTGGTCAAGTCGGC	205
	Antisense	CTTCCAATGTAGATCCCCAAT	

## Data Availability

The data supported this research will be made available upon request, which should be sent to the corresponding author.

## References

[1] Etz C. D., Weigang E., Hartert M. (2015). Contemporary spinal cord protection during thoracic and thoracoabdominal aortic surgery and endovascular aortic repair: a position paper of the vascular domain of the European Association for Cardio-Thoracic Surgery. *European Journal of Cardio-Thoracic Surgery*.

[2] Wang B., Zhu Q., Man X., Guo L., Hao L. (2014). Ginsenoside Rd inhibits apoptosis following spinal cord ischemia/reperfusion injury. *Neural Regeneration Research*.

[3] Yu D., Li M., Ni B., Kong J., Zhang Z. (2013). Induction of neuronal mitophagy in acute spinal cord injury in rats. *Neurotoxicity Research*.

[4] de Lavor M. S. L., Binda N. S., Fukushima F. B. (2015). Ischemia-reperfusion model in rat spinal cord: cell viability and apoptosis signaling study. *International Journal of Clinical and Experimental Pathology*.

[5] Wu H.-J., Pu J. L., Krafft P. R., Zhang J. M., Chen S. (2015). The molecular mechanisms between autophagy and apoptosis: potential role in central nervous system disorders. *Cellular and Molecular Neurobiology*.

[6] Feng Y., He D., Yao Z., Klionsky D. J. (2014). The machinery of macroautophagy. *Cell Research*.

[7] Xie L., Yu S., Yang K., Li C., Liang Y. (2017). Hydrogen sulfide inhibits autophagic neuronal cell death by reducing oxidative stress in spinal cord ischemia reperfusion injury. *Oxidative Medicine and Cellular Longevity*.

[8] Hao H.-H., Wang L., Guo Z. J. (2013). Valproic acid reduces autophagy and promotes functional recovery after spinal cord injury in rats. *Neuroscience Bulletin*.

[9] Wang L., Feng D., Liu Y. (2017). Autophagy plays a protective role in motor neuron degeneration following spinal cord ischemia/reperfusion-induced spastic paralysis. *American Journal of Translational Research*.

[10] Chen M., Li X., Fan R. (2018). Cadmium induces BNIP3-dependent autophagy in chicken spleen by modulating MiR-33-AMPK axis. *Chemosphere*.

[11] Bellot G., Garcia-Medina R., Gounon P. (2009). Hypoxia-induced autophagy is mediated through hypoxia-inducible factor induction of BNIP3 and BNIP3L via their BH3 domains. *Molecular and Cellular Biology*.

[12] Lin F., Pan L.-H., Ruan L. (2015). Differential expression of HIF-1*α*, AQP-1, and VEGF under acute hypoxic conditions in the non-ventilated lung of a one-lung ventilation rat model. *Life Sciences*.

[13] Wu H., Huang S., Chen Z., Liu W., Zhou X., Zhang D. (2015). Hypoxia-induced autophagy contributes to the invasion of salivary adenoid cystic carcinoma through the HIF-1*α*/BNIP3 signaling pathway. *Molecular Medicine Reports*.

[14] Schofield C. J., Ratcliffe P. J. (2004). Oxygen sensing by HIF hydroxylases. *Nature Reviews Molecular Cell Biology*.

[15] Rytkönen K. T., Williams T. A., Renshaw G. M., Primmer C. R., Nikinmaa M. (2011). Molecular evolution of the metazoan PHD-HIF oxygen-sensing system. *Molecular Biology and Evolution*.

[16] Kim S. Y., Yang E. G. (2015). Recent advances in developing inhibitors for hypoxia-inducible factor prolyl hydroxylases and their therapeutic implications. *Molecules*.

[17] Xu W.-L., Wang S. H., Sun W. B. (2019). Insufficient radiofrequency ablation-induced autophagy contributes to the rapid progression of residual hepatocellular carcinoma through the HIF-1*α*/BNIP3 signaling pathway. *BMB Reports*.

[18] Chinnadurai G., Vijayalingam S., Gibson S. B. (2008). BNIP3 subfamily BH3-only proteins: mitochondrial stress sensors in normal and pathological functions. *Oncogene*.

[19] Zhang J.-J., Zhang Z.-Z., Ke J.-J. (2014). Protection against intestinal injury from hemorrhagic shock by direct peritoneal resuscitation with pyruvate in rats,. *Shock*.

[20] DeBoer L. W., Bekx P. A., Han L., Steinke L. (1993). Pyruvate enhances recovery of rat hearts after ischemia and reperfusion by preventing free radical generation. *The American Journal of Physiology*.

[21] Ryou M.-G., Liu R., Ren M., Sun J., Mallet R. T., Yang S. H. (2012). Pyruvate protects the brain against ischemia-reperfusion injury by activating the erythropoietin signaling pathway. *Stroke*.

[22] Yu S., Xie L., Liu Z., Li C., Liang Y. (2019). MLN4924 exerts a neuroprotective effect against oxidative stress via Sirt1 in spinal cord ischemia-reperfusion injury. *Oxidative Medicine and Cellular Longevity*.

[23] Schievink W. I., Luyendijk W., Los J. A. (1988). Does the artery of Adamkiewicz exist in the albino rat?. *Journal of Anatomy*.

[24] Basso D. M., Beattie M. S., Bresnahan J. C. (1995). A sensitive and reliable locomotor rating scale for open field testing in rats. *Journal of Neurotrauma*.

[25] Cheng M.-C., Pan T.-M. (2017). Glyceryl 1,3-dipalmitate produced from *Lactobacillus paracasei* subspecies. *Paracasei* NTU 101 inhibits oxygen-glucose deprivation and reperfusion-induced oxidative stress via upregulation of peroxisome proliferator-activated receptor *γ* in neuronal SH-SY5Y cells. *Journal of Agricultural and Food Chemistry*.

[26] Hong S., Kwon J., Kim D. W., Lee H. J., Lee D., Mar W. (2017). Mulberrofuran G protects ischemic injury-induced cell death via inhibition of NOX4-mediated ROS generation and ER stress. *Phytotherapy Research: PTR*.

[27] Kalogeris T., Baines C. P., Krenz M., Korthuis R. J., Terjung R. (2016). Ischemia/reperfusion. *Comprehensive Physiology*.

[28] Kalogeris T., Baines C. P., Krenz M., Korthuis R. J. (2012). Cell biology of ischemia/reperfusion injury. *International Review of Cell and Molecular Biology*.

[29] Weaver J. L., Matheson P. J., Matheson A. (2018). Direct peritoneal resuscitation reduces intestinal permeability after brain death. *Journal of Trauma and Acute Care Surgery*.

[30] Bar-Yosef T., Damri O., Agam G. (2019). Dual role of autophagy in diseases of the central nervous system. *Frontiers in Cellular Neuroscience*.

[31] Kihara A., Noda T., Ishihara N., Ohsumi Y. (2001). Two distinct Vps34 phosphatidylinositol 3-kinase complexes function in autophagy and carboxypeptidase Y sorting in Saccharomyces cerevisiae. *The Journal of Cell Biology*.

[32] Maejima Y., Isobe M., Sadoshima J. (2016). Regulation of autophagy by Beclin 1 in the heart. *Journal of Molecular and Cellular Cardiology*.

[33] Liu Y., Eaton E. D., Wills T. E., McCann S., Antonic A., Howells D. W. (2018). Human ischaemic cascade studies using SH-SY5Y cells: a systematic review and meta-analysis. *Translational Stroke Research*.

[34] Li L., Jiang H.-k., Li Y.-p., Guo Y.-p. (2015). Hydrogen sulfide protects spinal cord and induces autophagy via MiR-30c in a rat model of spinal cord ischemia-reperfusion injury. *Journal of Biomedical Science*.

[35] Fang B., Li X.-Q., Bao N.-R. (2016). Role of autophagy in the bimodal stage after spinal cord ischemia reperfusion injury in rats. *Neuroscience*.

[36] Fan J., Zhang Z., Chao X. (2014). Ischemic preconditioning enhances autophagy but suppresses autophagic cell death in rat spinal neurons following ischemia-reperfusion. *Brain Research*.

[37] Meneses A. M., Wielockx B. (2016). PHD2: from hypoxia regulation to disease progression. *Hypoxia*.

[38] Wang F., Liang G.-Y., Liu D.-X. (2015). Effect of Si-RNA-silenced HIF-1*α* gene on myocardial ischemia-reperfusion-induced insulin resistance. *International Journal of Clinical and Experimental Medicine*.

[39] Chiara Maiuri M., Le Toumelin G., Criollo A. (2007). Functional and physical interaction between Bcl-X_L_ and a BH3-like domain in Beclin-1. *The EMBO Journal*.

[40] Li Y., Wang Y., Kim E. (2007). Bnip3 mediates the hypoxia-induced inhibition on mammalian target of rapamycin by interacting with Rheb. *The Journal of Biological Chemistry*.

[41] Chen B.-C., Weng Y. J., Shibu M. A. (2018). Estrogen and/or estrogen receptor *α* inhibits BNIP3-induced apoptosis and autophagy in H9c2 cardiomyoblast cells. *International Journal of Molecular Sciences*.

[42] Peng H., Kasada A., Ueno M. (2018). Distinct roles of Rheb and Raptor in activating mTOR complex 1 for the self- renewal of hematopoietic stem cells. *Biochemical and Biophysical Research Communications*.

[43] Robb K. P., Cotechini T., Allaire C., Sperou A., Graham C. H. (2017). Inflammation-induced fetal growth restriction in rats is associated with increased placental HIF-1*α* accumulation. *PLoS One*.

[44] Ren H., Liu N.-Y., Song X.-F., Ma Y.-S., Zhai X.-Y. (2011). A novel specific application of pyruvate protects the mouse retina against white light damage: differential stabilization ofHIF-1*α* andHIF-2*α*. *Investigative Ophthalmology & Visual Science*.

[45] D’Angelo G., Duplan E., Boyer N., Vigne P., Frelin C. (2003). Hypoxia up-regulates prolyl hydroxylase Activity. *The Journal of Biological Chemistry*.

[46] D’Angelo G., Duplan E., Boyer N., Vigne P., Frelin C. (2003). Hypoxia up-regulates prolyl hydroxylase activity: a feedback mechanism that limits HIF-1 responses during reoxygenation. *The Journal of Biological Chemistry*.

[47] Demidenko Z. N., Blagosklonny M. V. (2011). The purpose of the HIF-1/PHD feedback loop: to limit MTOR-induced HIF-1*α*. *Cell Cycle*.

